# Advancements in Pulse Starches: Exploring Non-Thermal Modification Methods

**DOI:** 10.3390/foods13162493

**Published:** 2024-08-08

**Authors:** Pranita Mhaske, Asgar Farahnaky, Mahsa Majzoobi

**Affiliations:** 1 AFB International, 3 Research Park Drive, St. Charles, MO 63304, USA; pvmhaske@afbinternational.com; 2 Biosciences and Food Technology, RMIT University, Bundoora West Campus, Plenty Road, Melbourne, VIC 3083, Australia; asgar.farahnaky@rmit.edu.au

**Keywords:** pulse starch, non-thermal modification, clean label ingredients, starch value addition

## Abstract

The surge in the global demand for plant-based proteins has catapulted pulse protein into the spotlight. To ensure economic viability and sustainable production, it is crucial to utilize pulse starch, a by-product of plant protein fractionation. Despite the increasing interest in pulse starches, there is a notable gap in knowledge regarding their modifications and applications compared to cereal and tuber starches. Non-thermal techniques such as electron beam radiation, static high pressure, microfluidization, and cold plasma are emerging as innovative methods for starch modification. These techniques offer significant advantages, including enhanced safety, environmental sustainability, and the development of unique functional properties unattainable through conventional methods. However, challenges such as equipment availability, high costs, and energy consumption hinder their widespread adoption. In light of the growing emphasis on “clean and green labelling” and effective “waste management” in food production, evaluating non-thermal techniques for pulse starch modification is critical. This review aims to thoroughly assess these non-thermal techniques and their combinations, offering valuable insights for researchers and the food industry. By maximizing the potential of pulse starches in innovative food applications, it provides a comprehensive guide for effective non-thermal methods that add value and align with sustainable practices.

## 1. Introduction

The dried edible seeds of leguminous crops, other than the ones used for oil extraction (e.g., peanut and soybean), are known as pulses. With roughly 60 domesticated species of pulses throughout the world, pulses are considered a staple food rich in complex carbohydrates, high-quality proteins, and bioactive compounds [1]. Globally, 71 million tons of pulses are produced annually, which are consumed as whole, split, and dehulled grain and/or flour. They are an affordable alternative to animal protein sources [2]. Due to the low protein value and lack of palatability of non-legume plant proteins, pulse proteins are the preferred source of plant proteins [3]. Dry or wet fractionation is widely used to produce pulse protein isolates/concentrates, which have found extensive applications in food products due to their unique functional and nutritional properties and are in high demand, in line with the recent food innovation trends for plant-based and sustainable products [4].

Pea, lentil, and faba bean are currently the most utilized pulse proteins in meat analogues [5]. While pulses are renowned for their high protein contents, starch constitutes the predominant component, accounting for between 35 and 60% of their composition [2]. Despite this, pulse starches are less available commercially, largely due to the complicated extraction and higher price, which has led to their relative neglect in comparison to other starch sources. As the demand for pulse proteins rises, there is a corresponding surge in interest in utilizing pulse starch, a by-product of pulse protein fractionation [5]. This trend is projected to drive growth in the pulse starch market, with an estimated annual growth rate of 4.85% from 2021 to 2028 [2]. Pulse starches have unique features compared to tuber and cereal starches, including a higher amylose content, longer amylopectin branched chains, and often are more resistant to thermal and shear treatments. This makes them suitable for numerous novel food applications such as the production of meat analogues, cooked sausages, extruded products, canned foods, vermicelli, noodles, soups, and sauces to improve their textural properties and are a good substitute for chemically modified starches, including cross-linked starches [3,6]. Additionally, pulse starches have lower digestibility (lower glycemic index) due to their molecular structure, high amylose content, and high tendency to retrograde [6]. Although, just like tuber and cereal starches, native pulse starches have functional drawbacks such as rapid retrogradation and subsequent poor cold-storage stability, which necessitate modification to enhance their nutritional and functional properties to promote widespread industrial applications.

Native starch is often modified to improve its functional properties and broaden its application in a wider range of products. Multiple strategies exist for creating customized modified starches, including chemical, biochemical, physical, enzymatic, genetic, and combinations of these methods [7]. Conventional chemical modification of starch has been widely used but has recently faced severe backlash due to its adverse ecological impact, chemical residues, waste treatment, cost of chemicals, and, more importantly, consumer concerns. Currently, there is a growing demand for innovative green and clean starch, and hence sustainable and echo-friendly modification methods [7,8]. Among these methods, non-thermal starch modifications, including radiation, high-pressure, electric fields, and cold plasma, hold promise for the development of functional starches tailored to meet diverse industrial and consumer demands [9]. The utilization of non-thermal methods offers several advantages, including safety, improved control over modification parameters, and reduced energy consumption. Modified starches produced with these innovative techniques exhibit distinct functional properties that conventional methods often cannot achieve. These properties encompass the creation of microporous materials, chemical-free emulsifiers, nano-starch particles, and starches suitable for crafting 3D foods [10,11,12,13]. Despite these benefits, some non-thermal methods are underutilized for starch modification due to factors like limited large-scale equipment availability, high costs, and energy consumption, as well as insufficient knowledge about optimal operating conditions and the resulting modified starches’ functional properties and applications [14,15]. Therefore, exploring the applications of novel non-thermal techniques for starch modification is crucial for bridging the knowledge gap and unlocking their full potential in terms of unique functional properties, economic feasibility, sustainability, health benefits, and diverse food applications.

Most commercially available modified starches are derived from primary sources like corn, wheat, cassava, and potato, with modification methods refined over decades of research and innovation. In contrast, pulse starch modification remains underexplored, with limited information on its structural impact, functional properties, and digestibility. As pulse starches continue to emerge, there is an increasing need for their modification to enhance their suitability for food production. Consequently, there is a concurrent need to gather information regarding modification strategies and the resultant new physicochemical properties.

In this review, a comprehensive analysis of articles published on the modification of pulse starches using non-thermal methods over the last decade was conducted. Studies combining a non-thermal method with either a thermal or a chemical method of modification were excluded to focus solely on emerging non-thermal techniques. This review article aims to consolidate findings from the relevant literature on the physicochemical characteristics and digestibility of pulse starches, creating a comprehensive repository of current insights. This information can streamline the processing of modified pulse starches and enhance their applications across both the food and non-food sectors.

## 2. Non-Thermal Technologies for Pulse Starches

**Figure 1:**
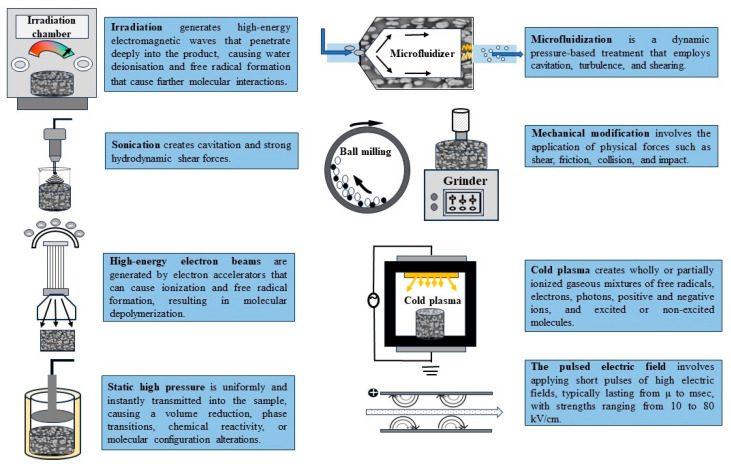
Innovative non-thermal technologies for starch modification and their mechanisms of action (this figure was produced by the authors).

Figure 1 illustrates the use of different non-thermal technologies for producing modified pulse starches. However, there are gaps and not all available methods have been tested on pulse starches. Therefore, this review focuses on the most studied methods. While the food applications of various modified starches produced using novel non-thermal methods have been explored (see Table 1), others remain to be studied. The following sections detail these methods and their effects on pulse starches, providing examples and discussing food applications.

### 2.1. Pulse Starch Modification Using Irradiation (IR)

Both the US Food and Drug Administration and international standards endorse the use of gamma rays from cesium-137 or cobalt-60. However, due to practical challenges in handling cesium-137, its industrial usage is rare. Modification of starch using gamma IR is gaining popularity as it is a rapid, cost effective, and non-toxic method of starch modification (Table 2). Gamma IR interacts with the water molecules present in starch, breaking them down into high energy electrons and short-lived free radicles (H and OH). These induce modification of the starch by either fragmentation or crosslinking in the presence of oxygen. Gamma rays can also directly break down glycosidic bonds at chain ends and cleave amylopectin branches, altering the physicochemical and functional properties of starch (e.g., variations in morphology, amylose content, chemical composition, crystallinity, enzymatic digestion, thermal and textural properties, etc.) [8,16]. An increase in solubility has been reported after the irradiation of starch from multiple sources, such as broad bean, chickpea, cowpea, and black, red, white, and yellow kidney beans, with a positive correlation with an increase in dosage. Additionally, a drop in swelling power was related to the cleavage of the glycosidic bonds and depolymerization of starch molecules, especially amylopectin [16,27,28,29].

The water absorption capacity showed an increasing trend with increasing dosage in broad bean and chickpea starch [16,28], while the swelling index had a negative correlation with the irradiation dosage [27,29]. Gamma IR also reduces the water holding capacity of the starches and can therefore be used to increase the shelf-life and reduce the degradation of starch during storage [27].

Broad bean, cowpea, and mung bean starches maintained their native C-type crystallinity, with a decrease in relative crystallinity [28,29,31]. IR partially changed the starch structure, resulting in smaller molecules and some changes in the granular morphology. Scanning electron microscopy (SEM) scans also revealed surface cracks on chickpea starch at elevated doses of IR [16]. The absorbance intensities of O-H and C-H stretches of irradiated chickpea starches show marked decreases that were most likely caused by the homolytic cleavage of the bonds induced by the free radicles generated in starch.

A reduction in pasting properties, most likely caused due to degradation/depolymerization and restricted swelling of starch granules caused by IR, has been reported in broad bean, kidney bean, and cowpea starch [27,28,29]. Upon cooling, irradiated mung bean starch pastes formed harder gels, as the cleavage of glycosidic bonds leads to the formation of molecules with a shorter chain length, which are more likely to re-associate on cooling [31].

Significant drops in gelatinization temperature, retrogradation and gelatinization enthalpy caused by partial depolymerization of branched amylopectin chains and cleavage of double helical bonds have been reported in chickpea, cowpea, and black, red, white, and yellow kidney bean starch [16,27,29,30].

The paste clarity of mung bean starch increased after IR. Carboxylic groups are electronegative and cause electrostatic repulsion between starch molecules and impair their association. With less intra- and intermolecular associations, the paste clarity improves due to increased transmittance [31]. A decrease in the L (lightness) value and an increased b (yellowness) value of irradiated alkali-extracted chickpea starch has been reported [16]. An increase in the antioxidant properties of cowpea starch measured as ferric reducing antioxidant power, FRAP values, and DPPH% inhibition after IR has been found. The increased antioxidant capacity is due to the new double bonds formed because of radiation-induced degradation, which reduces the free radical reactivity [29].

Overall, IR of starches can improve the cooking properties of starch while maintaining the starch quality, lowering syneresis, and improving freeze–thaw stability, making them suitable for applications in refrigerated and frozen food products [28]. IR has shown some potential for creating transparent starches without using chemicals.

### 2.2. Pulse Starch Modification Using Ultrasonication (US)

US is a physical treatment that uses high-power ultrasound at a low frequency (~15–40 kHz) to modify starch. In a starch–water dispersion, the formation, growth, and collapse of microbubbles in very short times is called as cavitation. Cavitation causes strong hydrodynamic shear forces that alter the structural and functional properties of starch [32]. The extent of the modification is controlled by various factors, including the frequency and intensity of ultrasound, temperature, water content of the system, starch type, and the duration of sonication (see Table 3 for some examples).

The effect of sonication on two legume starches, chickpea and lentil, was examined at 30% power and a 50% pulse for 15 min in an ice bath. The study revealed increases in swelling power and flow behavior index (n), along with decreases in the consistency index (K), solubility, apparent viscosity, gelatinization temperatures, NMR T2 relaxation times, and particle size. Sonication treatment facilitated granular dissolution, enhancing the gelatinization process and viscosity. While the FTIR spectra of the modified starches maintained their native peaks, there was a reduction in intensity. SEM micrographs indicated that sonication smoothed the surface of the granules but led to the development of cracks after treatment [37].

Carioca bean starch underwent modification through sonication (30 s on cycles, 5 s off cycles, at a 50% amplitude and 20 kHz frequency for 90 min). This resulted in improved pasting properties, while the thermal properties, size distribution, molecular structure of debranched starch fractions, and granular structure remained unaffected [38]. The increase in paste viscosity can be attributed to the increased water absorption facilitated by the cavitation during sonication that degrades the polysaccharide chains and/or weakens the amylose–amylopectin interaction. Sonication also resulted in a weaker physical structure that was susceptible to collapse with an increase in temperature or shear; this is in line with the higher breakdown value exhibited by sonicated starches [38]. A comparative investigation into the effects of sonication on pea and vetch starches revealed decreases in the amylose content, swelling index, syneresis, oil absorption capacity, and pasting properties. Conversely, there were increases in the emulsion capacity and water absorption capacity following sonication treatment. All physicochemical, functional, and pasting properties exhibited insignificant variations before and after sonication and between the two starch sources i.e., pea vs. vetch starch [39]. This similarity in the effect of the treatment can be attributed to the fact that the native pea and vetch starches were similar in their functional and physicochemical properties, inducing similar results upon treatment. Sonication also showed a drop in amylose content of lentil starches with insignificant decreases in syneresis and functional properties like the solubility and swelling indices, paste clarity, water and oil absorption capacities, emulsion capacity and stability, pasting properties, and crystallinity. FTIR spectra showed the formation of a new peak at 2938 cm^−1^ due to CH_2_ deformation. “L” values (lightness) decreased while “b” values (yellowness) increased [39]. Upon the sonication of mung bean starch, observations revealed some granular surface corrosion, although there were no alterations in the granular shape. Furthermore, the molecular weight, crystallinity, and amylopectin long chains decreased, while the amylose content, solubility, swelling power, gelatinization temperatures, enthalpy, and pasting viscosity increased [32]. These results are consistent with previous findings regarding the impact of sonication (300 W and 20 kHz for 20 min) on pea starch. Specifically, there was greater degradation of amylopectin, leading to a reduction in its molecular weight, while the molecular weight of amylose increased [40]. Microscopic examinations unveiled disruptions in the distribution of reducing ends and alterations in the structure of growth rings. Pasting properties, including peak, trough, final viscosity, breakdown, and setback, exhibited notable increases, aligning with the expected rise in the amylose content. This increase in the amylose content was corroborated by FTIR results, which indicated a significant decrease in the crystalline-to-amorphous ratio post-sonication. Swelling power and solubility showed increases with temperature up to 70 °C, followed by declines, which were possibly attributable to starch molecule depolymerization during modification. X-ray diffraction analysis revealed no changes in C-type crystallinity and only a reduction in relative starch crystallinity. Additionally, during an investigation into ultrasound’s efficacy in augmenting the resistant starch content in pea starch, observations included rough surfaces and granular cracks. Swelling power increased with temperature, while solubility remained constant and lower than that of native starch [41]. The physicochemical and functional attributes of the resultant starch are highly influenced by the starch type and the processing conditions. As a result, contradictory findings may occasionally emerge in the literature.

Non-chemical sonication treatment of starches is advantageous, with short processing times, enhanced yield and purity of the product, and reduced production costs, energy consumption, and environmental impacts. As sonication leads to the formation of cracks and pores on the granular surface, it can also be used as a pre-treatment to further facilitate starch modification by enzymatic/chemical/physical methods by enhancing reaction efficiency [9,15]. US can be used to produce nanostarch particles to be used in biodegradable films and as emulsifiers for Pickering emulsions [14,15].

### 2.3. Pulse Starch Modification Using High Hydrostatic Pressure (HHP)

HHP, also known as ultrahigh pressure, applies an immensely high pressure of 100–1000 MPa and more at temperatures below 50 °C for 2–30 min to starch slurries. The HHP process stands out for its environmental friendliness compared to thermal processes. Once the pressure is elevated, no additional energy consumption is necessary to maintain the pressure, eliminating the need for energy input to prevent pressure leakage [42,43].

Two key principles have been developed to describe the mechanism of HHP in food processing. The first is Le Chatelier’s principle that states that a decrease in volume caused by high pressure can cause changes such as phase transitions, chemical reactivity, or changes in molecular configuration. The second principle is the isostatic principle that asserts that pressure is uniformly and instantly transmitted, regardless of the size or geometry of the food [42]. A novel approach of using HHP is in pulse starch modification, which is shown in Table 3. Depending on the temperature, time, solvent and pressure applied, high-pressure modification can disrupt the granular structure; reduce crystallinity; increase the swelling and solubility of the starch granules; induce gelatinization; alter its thermal, pasting and rheological properties; and delay/decrease retrogradation and digestibility [42,43,44]. In addition to being a clean and non-thermal process, HHP offers advantages such as improved functionality, enhanced stability, and increased versatility in food and industrial applications [9,15]. Nevertheless, the primary drawbacks of this technology are associated with its high energy consumption, extended processing duration, and excessive pressure, all of which can lead to the destruction of starch granules [45].

In general, starch processing by HHP involves preparing a starch suspension in water (typically < 30%), which is then treated in an HHP vessel at the desired pressure (100–600 MPa) for a specific duration (≤30 min) at room temperature (20–30 °C). Subsequently, the pressure is rapidly released to atmospheric pressure (0.1 MPa) to disrupt non-covalent bonds. Finally, the pressurized starch suspension is air- or freeze-dried, milled, and stored at room temperature [22].

Lentil starch underwent HHP treatment for 10 min at 100, 400, 500, and 600 MPa. An increase in granule particle size was observed post-treatment and was attributed to damage to the outer section of the granules, which facilitated water absorption in the inner section, leading to swelling and aggregation of modified granules. Consequently, there was a noticeable rise in the water holding capacity. At 600 MPa for 10 min, the granules were completely gelatinized, as evidenced by rheometry, DSC, XRD, and SEM observations. Modified starch gels exhibited increased stiffness and a reduced dependence on pressure. High-pressure treatment transformed crystalline regions into amorphous ones. Peak viscosity, breakdown, and final viscosity notably decreased, while the pasting time increased. Granules treated at 600 MPa were completely destroyed, resulting in a larger particle size due to complete gelatinization. The crystallinity of native pea starch gradually shifted from the C-type to B-type pattern with increasing treatment pressure. At 600 MPa, the water absorption index, swelling power, and solubility increased between 30–70 °C and then declined. Pasting properties such as peak, trough, and final viscosity; breakdown; setback; gelatinization temperatures; and enthalpy decreased [33,46]. Similar findings were documented for pea starch dispersed in water or ethanol and subjected to a high pressure up to 600 MPa for 15 min. At 500 MPa, all samples were fully gelatinized, as evidenced by featureless DSC curves, while the particle size increased at 500 MPa before decreasing, possibly due to disintegration under pressure. Pasting properties increased with the treatment pressure up to 500 MPa before declining. Polarized light microscopy revealed a loss of birefringence, indicating the induction of “cold gelatinization” of pea starch dispersed in water. However, high-pressure treatment of starch dispersed in ethanol showed no discernible changes in starch properties [34]. Pea starch subjected to 500 MPa for 20 min exhibited a loss in granular shape and birefringence, along with a transition in crystallinity from A-type to B-type due to the retrogradation of pressure-treated starches. Consequently, there was an observed increase in gelatinization temperatures coupled with lower enthalpy, a trend consistent with other studies discussed in this context. Mung bean starch treated at varying pressures displayed increased storage and loss moduli, peaking at 480 MPa. This augmentation correlated with heightened rigidity and compactness. Pasting properties followed a similar trend but decreased at pressures higher than 480 MPa. These starch gels exhibited thixotropic pseudoplastic behavior.

In another study, high-pressure gelatinized mung bean starch showed gradual degradation of the Maltese cross pattern, with complete breakdown at 600 MPa. Native starch exhibited rapid swelling and solubility with heat, whereas pressurized starch showed a slower increase. Pressurized starch quickly recrystallized, restoring C-type crystallinity within 3 h. Recombination of leached amylose and amylopectin during retrogradation decreased light transmittance over time, while transition temperatures and retrogradation enthalpy increased.

Heat-gelatinized starch lacked a gelatinization peak during storage, indicating the absence of crystallization. Pressure-induced gelatinization likely involved incomplete disintegration of crystalline regions, stabilized by van der Waals and hydrogen bonds, leading to a distinct recrystallization mechanism from heat-induced gelatinized starch [47].

Red Adzuki bean starch, when exposed to HHP ranging from 0.1 to 600 MPa for 15 min, underwent complete gelatinization at 600 MPa. The treated starch exhibited a weakened diffraction peak intensity, reduced light transmittance, and decreased gelatinization temperature range and enthalpy, along with a lower pasting viscosity. Conversely, solubility, swelling power, pasting temperature, pasting time, gelatinization onset, and peak temperature increased with rising pressure. These alterations were ascribed to the structural disruption of granules induced by high-pressure treatment [47].

HHP can be used in the production of nanostarch, starch with emulsifying properties, highly soluble starch and pressure-induced gelatinized starch [14,48].

### 2.4. Pulse Starch Modification Using Microfluidization (MF)

MF, also referred to as high-pressure homogenization, is a dynamic pressure-based treatment that has yet to see extensive use in starch modification. This method involves passing starch slurries through a high-pressure homogenizer, which subjects them to high shear, turbulence, and cavitation while generating minimal heat during processing. Importantly, MF typically operates at lower pressures compared to hydrostatic high-pressure treatments [49]. As shown in Table 4, MF can cause pulse starch modification by inducing starch degradation at the granular and molecular levels, facilitating the intensification of physicochemical interactions and altering hydration properties, viscosity, solubility, the cation exchange capacity, bioavailability, and rheological properties [37,49]. It involves the disruption of covalent bonds within polymer chains by shear forces known as the mechanochemical action of homogenization. Dynamic pressure treatments can also induce complexation, starch esterification, and nanoparticle production [37,49,50]. Processing lentil and chickpea starches using a microfluidizer for five cycles at 130 MPa resulted in decreased solubility, particle size, apparent viscosity, span values, onset, and enthalpy of gelatinization, while the swelling power and T2 relaxation time increased. SEM micrographs indicated smoother surfaces with developed cracks on modified starch granules. The O-H stretching peak intensity at 3290 cm^−1^ increased, while characteristic peaks remained constant. Pea starch homogenized at 180 MPa for four cycles exhibited significant decreases in gelatinization temperature, enthalpy, and relative crystallinity, alongside increased retrogradation and a shift in crystallinity from C-type to B-type [36].

### 2.5. Pulse Starch Modification Using Cold Plasma (CP)

Plasma is often termed the fourth state of matter and denotes a state where increased internal energy transforms material from a solid to liquid to gas, and finally to an ionized gas state (plasma). CP arises from subjecting gas to high energies, typically through electric energy, which is recognized as the most convenient energy source [19,20]. The collision process within CP yields very small particle sizes, necessitating continuous energy input for its application. CP comprises wholly or partially ionized gaseous mixtures containing free radicals, electrons, photons, positive and negative ions, as well as excited or non-excited molecules. Plasma generation occurs under low-pressure or atmospheric pressure conditions, and is particularly applicable in the food processing industry. CP predominantly energizes electrons rather than heating the entire gas system, thereby maintaining gas molecules at room temperature, and hence is termed “cold plasma” or atmospheric cold plasma (ACP). This characteristic renders ACP suitable for food processing applications where thermal processing is undesirable. Plasma comprises unbound positive and negative particles with the net electrical charge being roughly zero [15,19,20,21].

Plasma species interact with starch through three possible mechanisms: cross-linking or grafting, depolymerization, and plasma etching. These mechanisms result in various surface modifications of biodegradable polymers. In addition to these mechanisms, introduced functional groups (such as carboxylic acid, peroxides, and hydroxyl groups) can also modify starch. Carbon dioxide gas plasma incorporates hydroxyls, ketones, aldehydes, and esters, while nitrogen and ammonia plasmas introduce primary, secondary, and tertiary amines through transformation mechanisms of these functional groups. Noble gas plasmas do not introduce functional groups but generate free radicals. However, aging effects like post-plasma rearrangement and post-plasma reactions should also be considered. The voltage applied, feed gas, and treatment time are the factors that determine the mode of starch modification [20,21].

There has been increasing interest in using the emerging CP processing method to modify starch functionality through interactions with the reactive plasma, and it has been used for various sources of starches; however, this technique has not been extensively utilized for pulse starch modification. The effect of plasma depends on the treatment time, voltage applied, and type of feed gas, with alterations in properties mainly caused by depolymerization and crosslinking of amylose and amylopectin chains [20].

CP treatment of mung bean starch preserved the granular shape but induced fissures, corrosions, and depositions on the surface. The amylose content initially rose with the treatment duration, indicating amylopectin degradation and subsequent amylose fragment formation. However, prolonged treatment led to amylose chain depolymerization. The amylose molecular weight increased while that of amylopectin decreased, suggesting susceptibility to CP-induced depolymerization. Crystallinity remained unchanged, but relative crystallinity dropped due to depolymerization. FTIR spectra showed no new peak formation, indicating no new chemical groups. Free radicals from plasma broke glycosidic linkages in the amylose double helix [32].

The solubility and swelling power of starches are affected by the molecular weight, chain length distribution of amylopectin, amylose–lipid complexes, amylose content, and interactions of amylose–amylopectin chains. The solubility of CP-treated starch was higher, as the partial depolymerization made the surface structure unstable and more susceptible to disruption during hydrothermal conditions. The pasting properties dropped with an increase in treatment time, as depolymerization decreased the interactions of gel components. On the other hand, after CP treatment, gelatinization temperatures and enthalpy increased with the treatment time for red Adzuki bean starch. This may be induced by crosslinking of amylopectin side chains caused by residual active plasma species, which stabilizes the crystallites that are not disintegrated during CP treatment [51].

### 2.6. Pulse Starch Modification Using Micronization

Micronization, an important technique, employs mechanical force to reduce the particle size to the micron-scale range. This method is applicable to both solid and liquid materials. Solid materials are typically processed through ball milling, grinding, jet milling, and colloid milling, whereas liquid materials are primarily micronized via high-pressure homogenization and microfluidization (described before) [52]. Among these methods, ball milling stands out as the most prevalent [52,53], while other techniques are less used for pulse starch modifications. When solid materials undergo mechanical modification, shear, high-pressure, friction, or other mechanical forces, mechanical energy becomes stored within them. This process generates numerous active sites, facilitating reactions with other reagents. The action of mechanical forces on solid materials typically progresses through three stages: stress, aggregation, and agglomeration. These mechanical forces can alter the energy landscape of chemical reactions, enabling new reaction pathways and providing a complementary synthetic strategy to conventional chemistry [33,43]. Consequently, mechanochemistry stands as an exciting and rapidly evolving field today [54]. Mechanical forces can modify the inherent properties of starch granules. Several active sites are produced that alter the reactivity of starches and can be tailored to mimic the effects of chemical modifications [53,54]. While it has been applied to other starch sources, mechanical modification has been less studied for pulse starch modification.

Mung bean starch underwent modification through grinding in a mortar machine for up to 48 h at pH 8–8.5. The native starch structure became blurry after 6–12 h, rough with depressions and bulges, and eventually disintegrated after 48 h. The initial C-type crystallinity decreased after 12 h, increased at 24 h, and then decreased again with continued grinding. FTIR spectra showed an increased intensity at 12 h, decreased intensity at 24 h, and then a consistent increase. NMR results indicated enhanced water mobility but a reduced binding capacity within the starch granules. The particle size initially increased, decreased at 24 h, and then increased again due to clumping, breakage, and subsequent adhesion. Gelatinization properties mirrored the particle size changes. Gelatinization temperatures and enthalpy decreased after 12 h, increased after 24 h, and then decreased again with continued grinding. Pasting properties decreased with prolonged treatment [54].

Various types of ball milling include planetary, tumbling, vibratory, and attrition mills, which have been elucidated previously [53]. This technique has been used to produce nanostarch particles and starch nanocrystals that are known as novel types of modified starches with applications as bioplastic filler in food packaging, food emulsifiers, fat replacers, antimicrobial agents, and encapsulating agents [12,18]. However, research on ball milling of pulse starches is scarce.

### 2.7. Pulse Starch Modification Using Electron Beam Irradiation (EBI)

EBI is an innovative method increasingly utilized for starch modification, driven by its eco-friendly profile with minimal by-product production and green labeling. Compared to other non-thermal methods, it offers advantages like higher throughput, flexibility, and the absence of radioactive waste. This technology involves subjecting starch samples to high-energy electron beams generated by electron accelerators like linear accelerators or microtrons. When these electrons interact with starch molecules, ionization occurs, resulting in the formation of free radicals. These radicals induce depolymerization of starch at the molecular level while preserving the granular structure at lower doses (<5 kGy). The technique has been instrumental in developing starch-based materials with improved functional properties such as enhanced solubility, thermal stability, and freeze–thaw stability [25,55]. Furthermore, this method has been found to increase the resistant starch content and influence the morphology and crystallinity of starch, albeit with a minimal impact on its microstructure. The characteristics of the treated modified starch vary depending on factors such as starch complexity, botanical origin, amylose content, crystallinity pattern, electron beam source, parameters, and frequency. Certain starch types, like B-type starches (e.g., yam, lotus, and potato), exhibit greater sensitivity to irradiation compared to A-type (e.g., rice) or C-type (e.g., bean) starches due to differences in granule interactions with electron beams [55]. Studies have shown that higher electron doses or multiple low-dose irradiations can modify starch properties without compromising its granular structure, thus conserving energy. Modified starch produced through EBI finds applications in creating stable starch gels and is widely used in beverages, liquids, or creamy systems pertinent to the cosmetic, pharmaceutical, and food industries.

Pea starch nanocrystals underwent electron beam-assisted pretreatment to explore its impact on the multiscale structure and physicochemical properties. It was found that the pretreatment did not alter the particle morphology, crystalline type, or FT-IR spectra of the starch nanocrystals. However, it did enhance the relative crystallinity, short-range orderliness index, solubility, apparent amylose content, zeta potential, and flow properties with increasing irradiation dose (5–20 kGy) [56].

It has been reported the surface of pea starch granules did not appear sensitive to irradiation treatment, even at high doses (under 30 kGy). The modification caused a significant reduction in the pea starch molecular weight that was attributed to E-beam-induced chain breakage of starch molecules. After the treatment, the crystal form of pea starch remained unchanged, indicating that irradiation did not affect the crystalline region of starch, but rather affected the amorphous region [56].

Following EBI of broad bean starch, the relative crystallinity and all pasting properties decreased, while solubility increased. These changes were attributed to free radical-mediated depolymerization of starch, which disrupts the crystalline structure. Additionally, the damage to amylopectin microcrystals could contribute to the decrease in relative crystallinity [26].

### 2.8. Pulse Starch Modification Using a Pulsed Electric Field (PEF)

PEF is a non-thermal processing technology that utilizes high-intensity electric fields with pulses ranging from microseconds to milliseconds. These pulses typically have amplitudes between 100–300 V/cm and 20–80 kV/cm, generated between two electrodes. PEF treatment is applied to food products for short durations, effectively inactivating microorganisms below the temperatures used in traditional thermal processing methods [24]. However, PEF has also been used for biopolymers and starch modification, as it significantly impacts macromolecules in food components, particularly enhancing the structure, viscosity, and gelatinization characteristics of starch. A PEF is applied on a starch slurry that requires adding electrically conductive salts to the starch slurry, which can lead to various effects, such as the introduction of carbonyl groups, degradation of glucans, and disintegration of starch granules. The effectiveness of a PEF for starch treatment may vary depending on factors like the electric field strength, treatment duration, and starch slurry concentration, as discussed in the following sections [23,24].

The impact of PEF treatment on the structural properties and digestibility of pea starch was investigated. It was found that PEF treatment altered the structure of starch granules, but did not affect X-ray diffractive patterns, fractal dimension, thickness of the semi-crystalline layered structure, or absorption peak positions in the infrared spectra. On the other hand, changes in the luminance of the Maltese cross, relative crystallinity, and molecular weight distribution of the treated starches were observed [57].

### 2.9. Pulse Starch Modification Using Combined Modifications

At present, the limitations of single modifications are increasingly unable to meet the growing demand for starch processing and hence the promotion of multiple modifications has occurred. To further improve the properties and uses of starches, combinations of various modification methods have been introduced.

A combination of sonication and gamma irradiation was used to modify lentil starch. It was found that the pasting properties of lentil starch, which were not affected upon sonication, were decreased significantly upon dual treatments. The amylose content of native starch showed a decrease upon sonication and dual treatments. Sonication and dual treatments decreased the Hunter “L” value, while “a” and “b” values showed increases. Syneresis, which was not affected by sonication, decreased significantly following dual treatments [39].

To explore the synergistic effects of EBI and ultra-high pressure on broad bean starch modification, varying pressures (200, 400, and 600 MPa) combined with different irradiation doses (3, 6, and 12 kGy) were employed. The results revealed that both treatments led to reductions in the amylopectin molecular weight and depolymerization of long chains, resulting in the loss of the short-range ordered structure and imperfections in the crystal structure. Additionally, starch viscosity, solubility, and enzyme sensitivity were improved by both treatments. Furthermore, the applied pressure altered the starch granule structure, while EBI facilitated further degradation and depolymerization by affecting both crystalline and amorphous regions. Thus, appropriate doses of EBI treatment can enhance the processing properties of ultra-high-pressure-modified starches, suggesting EBI as a promising method for promoting starch modification [56].

### 2.10. Effect of the Emerging Non-Thermal Modification on Digestion Properties of Pulse Starches

Starch, the primary carbohydrate in food, undergoes enzymatic breakdown in the small intestine by pancreatic α-amylase and glucosidase, yielding glucose [58]. The digestibility of starch is influenced by various factors, including its shape, size, presence of surface pores, amylose content, amylose/amylopectin ratio, crystalline type, interaction with other components, and processing or modification methods. Starches are categorized based on their digestibility into resistant starch (RS), slowly digested starch (SDS), and rapidly digested starch (RDS) [14,59].

Driven by consumer food and nutrition trends, there is a high food industry demand for novel starches that can be employed for engineering new foods with a reduced or controlled rate of glucose release. The effects of some of the emerging non-thermal starch modification techniques on pulse starch digestibility have been investigated, as shown in Table 4 and discussed below.

Gamma irradiation increased the RDS and RS contents of red, white, black, and yellow bean starch, while the SDS content dropped. The increase in the RDS content after irradiation is brought about due to the degradation of the granular and molecular structure that provides easy access to the digestive enzymes. The free radicles produced during irradiation are speculated to form stable matrices due to chain associations with increased resistance against digestive enzymes. This causes a decline in the SDS content, as it partially transforms into RS, increasing the RS content. During irradiation, cleaved glycosidic linkages induce mobility to the starch chains, which readily align to packed starch matrices, further boosting the RS content by being less susceptible to digestive enzymes [27].

Pea starch showed a drop in the hydrolysis degree when subjected to HHP. The hydrolysis degree dropped further with an increase in the treatment pressure, as did the RDS and SDS contents, while the RS content increased. These are most likely brought on by the compaction of molecules and interactions between amylose and amylopectin and the formation of B-type crystallinity with a reduced susceptibility to amylolytic enzymes [10].

Lentil starch exhibited a slight decrease in the resistant starch (RS) content when treated with pressures ranging from 400–500 MPa. However, an increase in the RS content was observed at 600 MPa. This rise was attributed to the elevated temperatures (38 °C) resulting from the adiabatic effect during pressurization. The combination of temperature and pressure led to the formation of more nuclei in starches, resulting in a higher quantity of recrystallized starch as resistant starch [33]. The in vitro digestibility of pea starches treated with HHP at 150, 300, 450, and 600 MPa for 25 min was lower compared to native pea starch (*p*  < 0.05) [46].

The efficacy of sonication in increasing the yield of RS3 (a subtype of resistant starch) in gelatinized pea starch was studied by investigating various parameters, including the US time (5, 10, 15, 20, and 25 min), pH (1, 3, 5, 7, and 9), and starch suspension concentration (15–65%) [41]. The highest yield was achieved at a 36% concentration with 13 min of sonication at pH 3.5, as optimized using response surface methodology. At concentrations above 35%, the starch paste became highly viscous, limiting water molecules’ access to the starch’s crystalline zones and hindering the complete disruption of hydrogen bonds. The RS3 content decreased with higher or lower pH values due to direct damage to the starch molecular structure at higher pH levels and increased hydrolysis at lower pH levels, impeding the formation of an ordered starch chain structure. Sonication was found to promote the orderly arrangement of starch chains and the formation of RS3. However, prolonged sonication led to direct disruption of the starch molecular structure.

HHP increased the RDS content but decreased the SDS and RS contents in broad bean starch. These were due to structural damage to granules, exposing more sites for enzyme action and accelerating hydrolysis. Pressure disrupted starch granules, destroying crystalline regions and increasing enzyme sensitivity, leading to a higher RDS content that peaked at 52.12% under the highest pressure. Additionally, pressure weakened interactions between amylose and amylopectin, reducing the RS content. EBI decreased the RDS content and increased SDS and RS contents, indicating reduced enzyme sensitivity and starch digestibility due to changes in the starch structure and chemistry, such as the formation of carboxyl groups and shorter amylopectin chains [25].

Pretreatment of pea starch by EBI significantly reduced the SDS + RS content, while increasing the RDS content [56]. Broad bean starch modified by EBI exhibited an increase in the RS content. This was attributed to the irradiation-induced depolymerization of amylopectin, resulting in the formation of more short chains (e.g., A chain), which form various degrees of aggregated structures, enhancing starch resistance. Additionally, EBI can induce the formation of carboxyl groups by interacting with digestive enzymes, thereby reducing their sensitivity and subsequently lowering starch digestibility [26]. In vitro digestion tests of PEF-treated pea starch showed that starch treated at relatively lower electric field intensities had an increased rapidly digestible starch content, reduced slowly digestible starch content, and decreased resistant starch content compared to the untreated starches [57].

## 3. Conclusions

The unique functional properties of pulse starches, including their low digestibility and strong gel formation, make them a valuable option in food manufacturing. The global trend towards reducing the carbon footprint in the food industry and adopting green and clean processing has compelled the starch industry to seek safe, sustainable, and chemical-free modification strategies. The non-thermal modification techniques reviewed here offer emerging opportunities to enhance the value of pulse starches, diversify their functionalities, and broaden their industrial applications. These techniques could result in the development of novel modified starches through physical modifications and their combinations without the use of hazardous chemicals.

Based on the current review of pulse starch modification over the last decade, it is evident that the utilization of advanced non-thermal modification methods for pulse starches is still in its nascent stage. Further research is essential to fully comprehend and unlock the potential of these innovative techniques and the modified pulse starches in food manufacturing. The most studied methods for pulse starch modification are high-pressure, sonication, and irradiation treatments, while other non-thermal methods, such as cold plasma, grinding, and EBI, and their combinations, are less investigated. Among various pulse starches, chickpea, lentil, pea, kidney bean, broad bean, and mung bean starches are commonly subjected to non-thermal modification, whereas research on the modification of other pulse starches is relatively limited. Further research is required to develop non-chemical and less resource-intensive processes for producing tailored modified starches for different food applications.

Typically, commercially available modified starches with high resistant starch levels (>70%) are developed using harsh chemical modification techniques, such as crosslinking with sodium trimetaphosphate (STMP) or high-temperature chloric acid dextrinizations. These starches, derived from maize, cassava, or wheat, are marketed as insoluble or soluble starch-based dietary fiber or resistant starch. Pulse starches containing higher levels of amylose compared to starches found in cereals and tubers are superior substrates for creating both soluble and insoluble dietary fiber based on starch.

The non-thermal technologies discussed in this review demonstrate significant potential to modify digestion properties and enhance resistant starch levels in pulse starches. Products derived from these novel and safer technologies could emerge as green, clean, health-promoting modified pulse starches. Further research is necessary to create new modified pulse starches with novel physicochemical properties using non-chemical technologies for formulating and designing safer foods. Furthermore, increasing the resistant starch content of emerging pulse starches to levels comparable to other commercially available starch-based dietary fiber deserves more research.

## Figures and Tables

**Table 1:** Main features and potential food applications of some emerging non-thermal technologies used for starch modification.

Emerging Non-Thermal Modification	Main Features	Potential Food Applications	Reference
Gamma irradiation	Must follow FDA regulations for food processing and labeling Uses dry starch for processing, with no need for further drying High efficiency Low cost No special appliances to control temperature No residues Easy to operate	Superabsorbent polymers used for biomaterials, drug delivery systems, and biodegradable films Increasing resistant starch under some processing conditions	[16]
Sonication	Easy to operate and control Simple to realize automation Short processing time No residues High product yield Applied on starch suspensions and requires further drying and milling A temperature elevation may occur due to heat generation Standardization and reproducibility are lacking	Production of microporous starch for the delivery of biomaterials Hydrogel production Emulsification Bio-absorbent material Encapsulation for controlled release materials Biofilm production Improving film stability Nanostarch particles	[14,17]
Micronization	Often needs a long processing time Both dry and wet applications High energy consumption Temperature may increase	Nanostarch particles Bioplastic filler in food packaging Food emulsifier Fat replacer Encapsulating agent for bioactive and antimicrobial materials Texture improver Gel agent	[12,18]
Cold plasma	Efficient Rapid Expensive Applied on dry starch	Nanoparticles Hydrogel Biofilm stability Reduces the hydrophilicity of starch films Low viscosity at high concentration	[19,20,21]
High hydrostatic pressure	Lower production cost compared to heat treatment Uses starch suspension for processing Requires further drying and milling	Edible and biodegradable films Hydrogels Fat replacer Production of low-calorie and low-GI foods Pressure-induced gelatinized starch Nanostarch	[22]
Microfluidization	Requires a starch suspension and further drying and milling Short and efficient Cost effective	Production of nanostarch Emulsifier Biofilms	
Pulsed electric field	Applied on starch suspensions and needs further drying and milling No residues Extremely fast Short time Requires a high-voltage electric field	Suitable for 3D printing of foods Improved solubility Increased sensitivity to digestive enzymes and chemical reactions for further modifications (i.e., multiple modifications)	[23,24]
Electron beam radiation	High throughput Extensive flexibility without radioactive waste Energy saving	Production of starch nanocrystals Encapsulation material Emulsification	[25,26]

**Table 2:** Modification of pulse starches using irradiation.

Starch Type	Modification Conditions	Parameters Evaluated	Findings	Reference
Chickpea	Source: Cobalt-60 Temp: 23 °C Doses: 0, 0.5, 1, 2.5, 5, and 10 kGy Rate: 0.5 kGy/h	Colour	Lightness decreased, yellowness and redness increased.	[30]
		Swelling and solubility index	The swelling index increased with the dosage at 50–70 °C, showed an irregular trend at 80 °C, and decreased at 90 °C. The solubility index increased with dosage and temperature.	
		Transmittance	Increased with dosage, decreased with temperature	
		Syneresis	Decreased with the dosage, increased with temperature	
		Pasting properties	RVA viscosity decreased with increasing dosage.	
		Thermal properties	Slight decrease	
		Morphology	Slight fissures were observed on the granule surface at higher dosages.	
		Crystallinity	The peak intensity decreased with the dosage.	
		Chemical composition	No changes	
		Water and oil absorption capacity	Increased	
Cowpea	Source: Cobalt-60 Doses: 0, 5, 10, 15, and 20 kGy	Swelling and solubility index	Swelling index decreased Solubility index increased	[29]
		Pasting properties	All properties decreased with an increase in the dosage.	
		Rheological properties	Yield stress, consistency index, and thixotropic breakdown decreased with the dosage; the flow behavior index increased.	
		Apparent amylose content	Decreased	
		Morphology	No significant changes	
		Crystallinity	Decreased	
		Thermal properties	All properties decreased with an increase in the dosage.	
		Antioxidant activity	DPPH inhibition increased FRAP increased	
Mung bean	Source: Cobalt-60 Temp: ~25 °C Doses: 0, 0.5, 1, 3, and 5 kGy	Morphology	Visible development of pores on the granular surface after treatment Some broken granules	[31]
		Crystallinity	Decreased	
		Molecular size distribution	Decreased with dosage	
		pH	pH was reduced with an increase in the dosage.	
		Pasting properties	All properties decreased with an increase in the dosage.	
		Gel strength	Increased	
		Water absorption and swelling index	The water absorption index remailed constant and the water swelling index fluctuated slightly with increases in the dosage and temperature.	
		Transmittance	Paste clarity increased with an increase in the dosage.	
Kidney bean	Source: Cobalt-60 Doses: 0, 5, 10, 15, 20, and 25 kGy Rate: 185 Gy/h	Solubility and swelling power	Solubility increased Swelling power decreased	[27]
		Carboxyl content	Increased	
		pH	Decreased	
		Light transmittance/retrogradation	Increased with the dosage Decreased upon storage	
		Apparent amylose content	Decreased	
		Amylose leaching	Increased with the dosage	
		Water absorption capacity	Increased with the dosage	
		Pasting properties	All parameters decreased with increasing dosages.	
		Thermal properties	All parameters decreased with increasing dosages.	
		Morphology	Dose-dependent surface cracking visible	
		Crystallinity	No significant change, except a slight decrease in intensity	
		Antioxidant activity	The DPPH radical scavenging property and ferric reducing/antioxidant power increase with the dosage.	

**Table 3:** Modification of pulse starches by high pressure.

Starch Type	Type of Modification	Modification Conditions	Parameters Evaluated	Findings	Reference
Lentil	High hydrostatic pressure	Starch/water = 1:4 Pressure: 400, 500, and 600 MPa Time: 10 min Compression rate: 4.2 MPa/s	Damaged starch	Increased	[33]
			Water interactions	Increased	
			Colour	Colour parameters were reduced	
			Morphology	No changes in surface morphology up to 500 MPa, visible changes on the starch surface with cracks at higher pressure	
			Particle size distribution	Increased	
			Pasting properties	RVA viscosity decreased	
			Thermal properties	No peak observed at 600 MPa	
			Crystallinity	Loss of crystallinity at 600 MPa	
			Chemical composition	Destruction of the crystalline structure of granules	
			Rheological properties	Improved starch gelatinization and G’ with pressure	
Pea	High hydrostatic pressure	Pressure: 300, 400, 500, and 600 MPa Time: 15 min Temp: 25 °C	Morphology	Irregular-shaped granules with a loss of birefringence observed at 600 MPa	[34]
			PSD	No change up to 400 MPa and then increased	
			Thermal properties	Insignificant changes	
			Pasting properties	RVA viscosity increased	
			Visual observation of dispersion	No changes at 400 MPa, with a higher sediment phase at 500 MPa A gel phase was observed at 600 MPa.	
			Recrystallization	Lower relative crystallinity after recrystallization	
			Light transmittance	Decreased	
			Thermal properties	Gelatinization temperature increased	
			Water solubility and swelling power	Both increased	
Mung bean starch	High hydrostatic pressure	Starch suspension: 20% Pressure: 120, 240, 360, 480, and 600 MPa Time: 30 min Temp: 25 °C	Pasting properties	Peak viscosity and pasting temperature increased with the increase in pressure.	[35]
			Power law consistency coefficient	Increased	
			Flow behavior index	Decreased up to 480 MPa and then increased	
			Apparent viscosity with shear rate	Decreased slightly at 120 MPa, increased up to 480 MPa, and then dropped lower than that of native starch Decreased with the increase in shear rate	
			Hysteresis loop	Increased	
Pea	Microfluidization	Suspension: 10% Pressure: 180 MPa Cycles: 4 cycles, 3 min each	Thermal properties	Decreased	[36]
			Retrogradation	Increased	
			Crystallinity	Relative crystallinity decreased Crystallinity changed from C-type to B-type	
Lentil	Microfluidization	Suspension: 6% Pressure: 130 MPa Cycles: 5 cycles	SP and Solubility	Solubility decreased Swelling power increased	[37]
			Rheological properties	The flow behavior index remained constant. The consistency index increased.	
			Morphology	The surface was smoothed, while granules developed cracks.	
			Chemical composition	No changes in native peaks, except an increased peak intensity	
			Size distribution	Decreased	
			Thermal properties	Decreased	
			T_2_ relaxation time	Increased	
Chickpea	Microfluidization	Suspension: 6% Pressure: 130 MPa Cycles: 5 cycles	Swelling power and solubility	Decreased	[37]
			Rheological properties	The flow behavior index increased. The consistency index decreased,	
			Morphology	The surface was smoothed, while granules developed cracks.	
			Chemical composition	No changes in native peaks, except an increased peak intensity	
			Size distribution	Decreased	
			Thermal properties	Decreased	
			Relaxation time	Increased	

**Table 4:** Digestion studies of modified pulse starches.

Type of Starch	Types of Modification	Modification Condition	Parameters Evaluated (%)	Findings	Reference
Lentil	High hydrostatic pressure	Starch/water = 1:4 Pressure: 400, 500, and 600 MPa Time: 10 min Compression rate: 4.2 MPa/s Decompression rate: 40 MPa/s	RS content	The resistant starch content changed from 5.07 to 4.47, 3.51 and 6.80% at 400, 500 and 600 MPa.	[33]
Pea	Sonication	Starch solutions: 15, 25, 35, 45, 55, and 65% pH: 1, 3, 5, 7, and 9 Temp: 90 °C Time: 5, 10, 15, 20, 25 min Power: 300 W Frequency: 100 kHz	RS3 content	The maximum RS3 content (~35.26%) was achieved at pH 3 when a 36% starch slurry was sonicated for 10 min.	[41]
Pea	High hydrostatic pressure	Starch suspension: 15% Pressure: 150, 300, 450, and 600 MPa Time: 25 min Temp: 30 °C	Starch fraction, hydrolysis degree	The % hydrolysis decreased with an increase in pressure. RDS was reduced from 58.9 to 52.4%; RS increased from 24.1 to 36.6% at 600 MPa.	[10]
Pea	Electron beam irradiation	0, 3, 6, and 12 kGy	RS, SDS, RDS	RDS was reduced from 44.41% to 38.66%; SDS increased from 9.74% to 12.75%; and RS increased from 45.85% to 48.59%.	[26]

## Data Availability

No new data were created or analyzed in this study. Data sharing is not applicable to this article.
